# Enhancement of Rheological Performance and Smoke Suppression in Sepiolite-Modified Asphalt

**DOI:** 10.3390/ma18245627

**Published:** 2025-12-15

**Authors:** Yongle Xu, Hongling Fan, Jing Yang, Peng Yin

**Affiliations:** 1 Dalian Construction Engineering Quality Testing Center Co., Ltd., Dalian 116023, China; 2 School of Infrastructure Engineering, Dalian University of Technology, No. 2, Linggong Road, Ganjingzi District, Dalian 116024, China; 13165155201@163.com (H.F.);

**Keywords:** modified asphalt, sepiolite, rheological properties, smoke suppression, microscopic interaction mechanism

## Abstract

To address the technical bottleneck of the coordinated improvement of high-temperature rutting resistance, low-temperature cracking resistance and environmental protection performance of road asphalt, and to address the existing problems in the research of sepiolite modified asphalt, such as the ambiguous microscopic mechanism of action, the lack of quantitative relationship between dosage and performance, and the unclear adaptability of modification processes, this study employed high-purity sepiolite as a modifier. After optimizing its microstructure through organic and surface modification, the sepiolite with the best compatibility with asphalt was selected. Four dosage gradients of 2%, 4%, 6%, and 8% were designed. Rheological tests were conducted to investigate the effects of sepiolite on the rutting resistance at high temperature, the cracking resistance at low temperature, and the fatigue durability of asphalt. Gas chromatography–mass spectrometry (GC–MS) was used to analyze changes in the organic components of asphalt fumes, while Fourier-transform infrared spectroscopy (FTIR) and gel permeation chromatography (GPC) were applied to reveal the microscopic interaction mechanisms and smoke-suppression principles. Results show that pristine sepiolite exhibits the best compatibility with asphalt. Although modified sepiolite shows a 43–45% increase in specific surface area, the overall high–low temperature coordination of the modified asphalt decreases by 10–15%. The sepiolite dosage has a significant influence on asphalt performance: when the dosage is 4–6%, the rutting factor of asphalt increases by 25–30%, indicating the best high-temperature deformation resistance; at 4%, the asphalt creep stiffness decreases by over 15%, minimizing the low-temperature cracking risk; and at 2–4%, the fatigue life extends by 9–13%, with the most notable improvement at 2%. In terms of smoke suppression, the porous structure of sepiolite adsorbs 3–5% of the light volatile components in asphalt, while its metal oxides inhibit the release of aliphatic and aromatic hydrocarbons, reducing toxic fume emissions by 12–18%. Microscopically, the interaction between sepiolite and asphalt is dominated by physical adsorption without chemical functional group recombination. The fibrous network of sepiolite enhances the structural stability of asphalt, while the adsorption of small and medium molecular components optimizes the molecular weight distribution, achieving a dual effect of performance enhancement and smoke suppression.

## 1. Introduction

In the modernization of transportation infrastructure, asphalt pavements have become the dominant pavement type in highway construction due to their superior driving comfort, low noise generation, and convenient construction. By the end of 2024, China’s total highway mileage exceeded 5.5 million kilometers, with asphalt pavements accounting for more than 75% of the total and over 90% of high-grade highways. Consequently, their service performance directly determines the efficiency and durability of the national road network [1,2]. However, conventional matrix asphalt exhibits significant performance limitations: it is prone to viscoelastic deformation and rutting under high temperatures, brittle cracking under low temperatures, and insufficient fatigue resistance under long-term traffic loading. These deficiencies severely constrain the long-term service stability of asphalt pavements [3,4]. In addition, asphalt must undergo heating at 150–180 °C during mixing, transportation, and paving, which promotes the volatilization of light components and the formation of complex fumes containing polycyclic aromatic hydrocarbons (PAHs), benzene series, and other volatile organic compounds (VOCs). These emissions not only pose irreversible respiratory hazards to construction workers but also exacerbate regional air pollution, creating a marked conflict with the green and low-carbon objectives of modern road engineering under the “dual-carbon” policy framework [5,6].

Furthermore, the high-temperature operations involved in asphalt pavement construction pose serious environmental and health concerns. The light fractions in conventional matrix asphalt—mainly low-carbon alkanes and aromatic compounds—volatilize extensively at elevated temperatures, producing complex fumes containing polycyclic aromatic hydrocarbons (PAHs), benzene derivatives, and other volatile organic compounds (VOCs). Among these, PAHs are highly carcinogenic; prolonged exposure can increase the incidence of respiratory diseases among construction workers by two to three times. Additionally, these emissions exacerbate regional ozone formation and elevate PM2.5 concentrations, creating a pronounced conflict with the national “dual-carbon” strategy and the principles of green transportation development [7,8,9]. Currently, commonly used fume-control techniques—such as combustion and condensation—primarily target end-of-pipe treatment. However, they suffer from major drawbacks, including high equipment costs, significant energy consumption, and the risk of secondary pollution, making them unsuitable for large-scale asphalt pavement construction [10,11].

To address the performance deficiencies and environmental issues of conventional asphalt, extensive research has been conducted on asphalt modification technologies. Early studies were dominated by polymer modification, which enhances both high- and low-temperature properties by forming interpenetrating networks; however, such approaches suffer from high cost, poor compatibility, and complex construction processes [12,13,14]. Fiber modification can improve the structural stability of asphalt mixtures but contributes little to the rheological improvement of the asphalt binder and fails to mitigate fume emissions [15,16]. In recent years, clay minerals have attracted growing attention owing to their large specific surface area, excellent stability, and low cost. For instance, montmorillonite can enhance high-temperature rutting resistance but often compromises low-temperature ductility [17,18], while kaolinite improves aging resistance yet shows limited effect on fatigue durability [19,20]. Most existing studies focus on improving a single performance aspect, with insufficient attention to the synergistic relationship between performance enhancement and fume suppression. Consequently, a comprehensive theoretical framework describing their interaction mechanisms has yet to be established.

Sepiolite, a natural fibrous silicate mineral, possesses a crystal structure composed of alternating silicon–oxygen tetrahedral sheets and magnesium–oxygen octahedral layers. Its unique one-dimensional nanofibrous morphology (0.2–10.0 μm in length) and continuous channel system distinguish it from conventional layered clays, endowing it with excellent mechanical reinforcement potential and strong molecular adsorption capacity [21,22,23]. In polymer composites, sepiolite can form a three-dimensional fibrous network that enhances the tensile strength of the matrix by 25–40% and increases the thermal decomposition temperature by 30–50 °C [24,25]. In environmental applications, its porous structure offers an adsorption capacity for organic pollutants 1.2–1.8 times higher than that of activated carbon, while its metal oxide components further improve pollutant removal efficiency through chemisorption [26,27]. These characteristics provide a versatile foundation for applying sepiolite in asphalt modification. From a performance perspective, the fibrous structure can form a physical skeleton within asphalt, restricting viscous flow at high temperatures and improving deformation coordination at low temperatures. From an environmental perspective, its porous channels can adsorb light volatile fractions, while metal oxides can inhibit the release of aromatic hydrocarbons, enabling a synergistic improvement in both performance and environmental sustainability [28,29].

Despite the promising potential of sepiolite in material modification, its application in asphalt engineering still faces several critical challenges. First, systematic comparisons of organic and surface modification processes are lacking, and the relationship between microstructural optimization of sepiolite and the enhancement of asphalt performance remains unclear. Second, the influence of sepiolite dosage on the multiple performance characteristics of asphalt has not been quantitatively investigated, and the determination of the optimal dosage range lacks an integrated evaluation framework. Third, studies on the smoke-suppression mechanism remain limited, as the synergistic effects between the porous structure and metal oxides have not yet been verified through microscopic analyses. Fourth, the engineering applicability of sepiolite-modified asphalt, including storage stability and construction workability, has not been systematically assessed, which constrains the practical implementation of laboratory research outcomes in large-scale engineering projects.

Based on the above research status and engineering needs, this study aims to achieve a dual objective of performance enhancement and fume reduction for conventional asphalt. A comprehensive theoretical and experimental investigation was conducted on sepiolite-modified asphalt. The microstructure of sepiolite was regulated through organic and surface modification, and the most compatible type was selected using physical property and rheological tests. Four dosage gradients (2%, 4%, 6%, and 8%) were designed to quantitatively evaluate the effects of sepiolite on the rutting resistance, low-temperature cracking resistance, and fatigue behavior of asphalt through rheological experiments. GC–MS was employed to analyze the variation in fume components, while FTIR and GPC were used to reveal the microscopic interaction mechanisms and smoke-suppression principles. The findings are expected to provide theoretical support and technical guidance for the large-scale application of sepiolite in asphalt modification for road engineering, promoting the development of high-performance, low-pollution, and cost-efficient green asphalt pavement materials.

## 2. Materials and Methods

### 2.1. Raw Materials

#### 2.1.1. Asphalt

A commonly used paving-grade asphalt was selected as the base binder for this study. Its technical properties conform to the specifications of Grade 70 matrix asphalt as defined in JTG E20-2011 Standard Test Methods of Bitumen and Bituminous Mixtures for Highway Engineering [30]. It should be noted that in the asphalt penetration grade system, the penetration range of grade 70 asphalt, 60.0–80.0 (0.1 mm), serves as the core basis for its classification. This interval is defined as follows: under the standard test conditions of 25 °C, 100 g load, and 5 s, the depth range of the standard needle penetrating the asphalt sample is 60 to 80 (0.1 mm). To ensure test repeatability and data reliability, all physical parameters were determined using standardized testing procedures, and the results are summarized in Table 1. The asphalt exhibited a penetration value of 64.2 (0.1 mm), which falls within the standard range of 60.0–80.0 (0.1 mm), indicating moderate hardness and viscosity at room temperature. Its softening point was 47.8 °C, satisfying the technical requirement of ≥46.0 °C, demonstrating satisfactory high-temperature stability. The ductility at low temperature exceeded 100 cm, reflecting good flexibility under cold conditions. From the perspective of chemical composition, the contents of saturates, aromatics, resins, and asphaltenes were 12.5%, 30.2%, 38.8%, and 18.5%, respectively. The relatively high proportions of aromatics and resins provide a favorable matrix environment for the physical adsorption and dispersion of sepiolite. However, they also result in typical performance deficiencies of conventional asphalt—namely, susceptibility to deformation at high temperatures and cracking at low temperatures—which necessitate performance enhancement through sepiolite modification.

#### 2.1.2. Sepiolite and Modification Treatment

**Figure 1:**
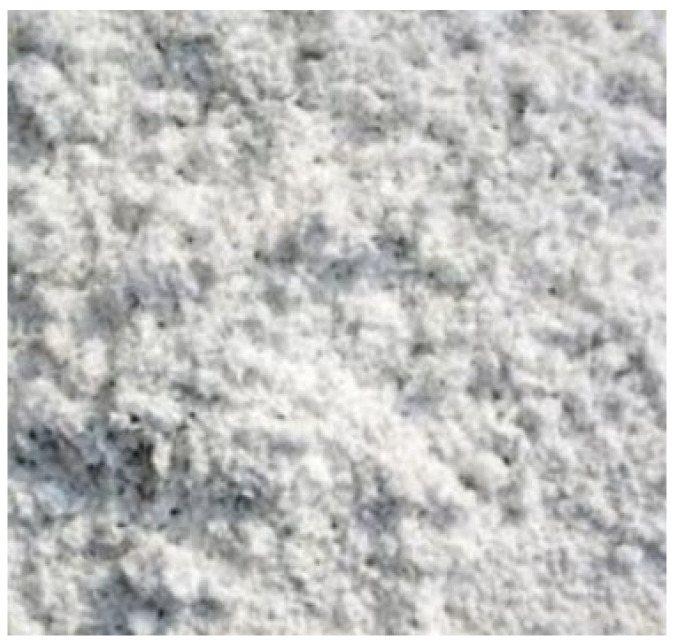
The appearance of sepiolite.

High-purity natural sepiolite was used in this study, with a mineral phase purity exceeding 98%. The material appears as white fibrous crystals, as shown in Figure 1. The particle size distribution, determined using a laser particle size analyzer, showed a 99.2% passing rate through a 325-mesh sieve (approximately 44 μm). The fiber length ranged from 0.2 to 10.0 μm, with an average aspect ratio of 25:1. This excellent fibrous morphology provides a solid basis for mechanical reinforcement within the asphalt matrix. The chemical composition of sepiolite was analyzed using X-ray fluorescence spectroscopy (XRF) (XRF-1800) from Shimadzu Corporation of Kyoto, Japan, and the results are presented in Table 2. SiO_2_ was the predominant component, forming the silicate tetrahedral framework of the sepiolite structure. MgO accounted for 24.15%, and together with Al_2_O_3_, it serves as the main metal oxide component capable of suppressing asphalt fume formation through chemisorption. Other components were present in minor quantities and had limited influence on the core properties of sepiolite.

To investigate the influence of microstructural optimization of sepiolite on the modification performance of asphalt, two treatment processes—organic modification and surface modification—were applied to the raw sepiolite. Based on relevant research experience [21,31], for the organic modification, a silane coupling agent was used as the modifier. An ethanol solution with a mass fraction of 2.5% was prepared, and the sepiolite was mixed with the solution at a mass ratio of 1:5. The mixture was stirred in a constant-temperature water bath at 50 °C and 300 r/min for 3 h to ensure that the alkoxy groups of the coupling agent fully reacted with the hydroxyl groups on the sepiolite surface, introducing hydrophobic functional groups to enhance interfacial compatibility with asphalt. After stirring, the mixture was vacuum-filtered, and the residue was dried in a vacuum oven at 80 °C for 4 h until a constant weight was achieved, yielding the organically modified sepiolite. For the surface modification, sodium dodecylbenzenesulfonate (SDBS) was employed as the surfactant. A 4% aqueous solution was prepared, and the sepiolite was mixed with the solution at a mass ratio of 1:4. The mixture was stirred at 40 °C and 250 r/min for 2 h, during which the hydrophilic groups of SDBS interacted electrostatically with surface charges on the sepiolite, while its hydrophobic chains enhanced affinity with asphalt molecules, improving dispersion within the asphalt matrix. After stirring, the mixture was filtered under reduced pressure and dried to a constant weight, obtaining the surface-modified sepiolite.

### 2.2. Methods

**Figure 2:**
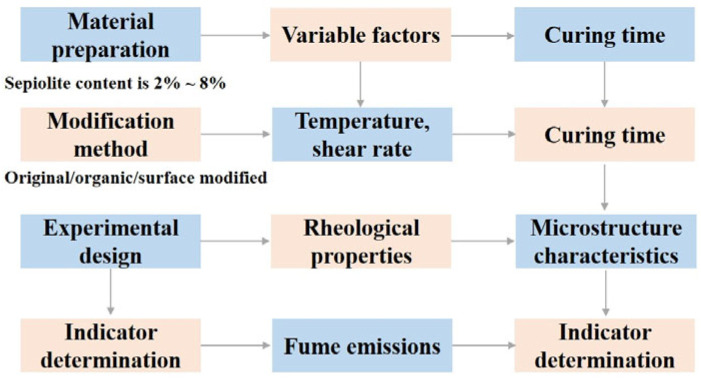
Research plan diagram.

The research plan of this study is shown in Figure 2.

#### 2.2.1. Characterization of Sepiolite Microstructure

To characterize the microstructural features of sepiolite before and after modification, the specific surface area and pore size distribution of raw sepiolite (RawSep), organically modified sepiolite (OSep), and surface-modified sepiolite (SSep) were measured using the liquid nitrogen adsorption–desorption method (BET–BJH analysis). Prior to testing, all sepiolite samples were vacuum pretreated at 100 °C for 6 h (heating rate 1 °C/min) to remove surface-adsorbed moisture and impurities. During the test, liquid nitrogen was used as the adsorbate, and adsorption–desorption isotherms were obtained at 77 K. The specific surface area was calculated according to the Brunauer–Emmett–Teller (BET) model, while the Barrett–Joyner–Halenda (BJH) model was applied to analyze the mesoporous (2–50 nm) pore size distribution and pore volume, thereby quantifying the influence of the modification processes on the microstructural characteristics of sepiolite.

#### 2.2.2. Preparation of Sepiolite-Modified Asphalt

Sepiolite-modified asphalt was prepared using a high-speed shear mixing method. The detailed procedure was as follows: First, the matrix asphalt was heated in an oven at 150 °C for 2 h to achieve a fully molten and uniform flowable state, while avoiding overheating to prevent thermal aging. Second, sepiolite (RawSep, OSep, or SSep) was added into the molten asphalt according to the designed dosages of 2%, 4%, 6%, and 8% by asphalt weight. The mixture was manually stirred for 5 min to ensure preliminary blending. Next, the mixture was placed in a 150 °C thermostatic water bath and subjected to high-speed shearing at 3500 r/min for 30 min using a high-shear mixer. During the process, the asphalt temperature was continuously monitored with a temperature sensor to prevent local overheating and degradation of the asphalt properties. After shearing, the modified asphalt was poured into molds preheated to 150 °C and then allowed to cool naturally to room temperature. Depending on the sepiolite type and dosage, the modified asphalts were denoted as RawSep-x%, OSep-x%, and SSep-x% (x = 2, 4, 6, 8), while the unmodified matrix asphalt served as the control sample.

#### 2.2.3. Physical and Rheological Property Tests

The physical property tests were conducted in accordance with the JTG E20-2011 Standard Test Methods of Bitumen and Bituminous Mixtures for Highway Engineering [30]. The ductility test was performed at 15 °C with a stretching rate of 5 cm/min, and each sample was tested in triplicate to obtain the average value. The softening point test was carried out with a heating rate of 5 °C/min, three times in parallel, and the temperature at the ball drop point was used to characterize the high-temperature deformation resistance of asphalt.

The rheological properties were evaluated using a dynamic shear rheometer (DSR) (Kinexus DSR-III) from Earth Products China Limited in Hong Kong, China, and all rheological test methods were carried out in accordance with the specification requirements of JTG E20-2011 [30]. In the temperature sweep test [32,33,34], the temperature ranged from 46 °C to 70 °C, with an angular frequency of 10 rad/s and a strain amplitude of 12%. The rutting factor (G/sinδ*) and phase angle (δ) were recorded to assess the high-temperature rutting resistance. The multiple stress creep recovery (MSCR) test was performed according to the AASHTO TP 70 standard at 64 °C [35], applying stress levels of 0.1 kPa and 3.2 kPa sequentially. The creep recovery rate (R) and non-recoverable creep compliance (Jnr) were calculated to evaluate the resistance to permanent deformation [36,37].

The bending beam rheometer (BBR) test was conducted following JTG E20-2011 and AASHTO T 313 [30,38], at test temperatures of −12 °C and −18 °C, recording creep stiffness (S) and creep rate (m). The ratio k = S/m was used to characterize the low-temperature cracking resistance [39,40].

The linear amplitude sweep (LAS) test was performed at 25 °C, consisting of a frequency sweep (strain 0.1%, frequency range 0.1–30.0 Hz) followed by a linear amplitude sweep (strain 0.1–30.0%, duration 310 s). Based on the viscoelastic continuum damage (VECD) model, the fatigue life (Nf) was calculated to evaluate the resistance of asphalt to fatigue damage [41,42,43].

#### 2.2.4. Analysis of Asphalt Fume Components

The smoke-suppression performance of asphalt was evaluated using a GC–MS system. Approximately 400 g of asphalt was placed in a heating furnace and maintained at 160 °C for 1 h. Air was introduced at a flow rate of 50 mL/min, and the generated fumes were absorbed by 100 mL of cyclohexane before being filtered through a 0.22 μm membrane. The GC–MS analysis was performed using a DB-5MS capillary column, with high-purity helium as the carrier gas. The temperature program was as follows: the column was initially held at 50 °C for 0.25 min, then heated to 150 °C at 3 °C/min and held for 2 min, followed by heating to 280 °C at 20 °C/min and maintained for 5 min. The relative contents of aliphatic and aromatic hydrocarbons in the fume were quantified using the peak area normalization method.

#### 2.2.5. Analysis of Microscopic Interaction Mechanisms

FTIR and GPC were employed to investigate the microscopic interaction mechanisms between sepiolite and asphalt [44,45]. For the FTIR test, the scanning range was 400–4000 cm^−1^ with a resolution of 4 cm^−1^. The absorption peaks of functional groups in the matrix asphalt and 4% RawSep-modified asphalt were compared to identify the dominant interaction types. For the GPC test, tetrahydrofuran (THF) was used as the mobile phase at a column temperature of 35 °C, employing a MIXED-C chromatographic column from Thermo Fisher Scientific in Waltham, MA, USA. Asphalt solutions with a concentration of 10 mg/mL in THF were filtered through a 0.22 μm membrane before injection. The relative proportions of different molecular weight components and the polydispersity index (PDI) were calculated to analyze the variations in molecular weight distribution.

## 3. Results and Discussion

### 3.1. Characterization of Sepiolite Microstructure

The microstructural parameters of RawSep, OSep, and SSep were determined using the BET–BJH method, and the results are presented in Table 3. As shown in the table, the modification processes significantly affected the specific surface area, pore volume, and pore size distribution of sepiolite. The BET specific surface area of RawSep was 107.95 m^2^/g, with a total pore volume of 0.32 cm^3^/g and an average pore diameter of 11.8 nm. After organic modification, the specific surface area of OSep increased to 154.83 m^2^/g, and the total pore volume rose to 0.45 cm^3^/g, while the average pore diameter decreased to 9.2 nm. This reduction can be attributed to the grafting of silane coupling agents onto the sepiolite surface, forming nanoscale protrusions that increase the number of active surface sites while refining the pore structure. For SSep, the specific surface area reached 156.53 m^2^/g, with a total pore volume of 0.48 cm^3^/g and an average pore diameter of 9.5 nm. The surface coating effect of sodium dodecylbenzenesulfonate (SDBS) further expanded the pore volume, though its influence on pore refinement was weaker than that of the organic modification. From the perspective of pore size distribution, mesopores (2–50 nm) accounted for more than 90% of the total pore volume in all three types of sepiolite. The proportions of mesopores in the 2–20 nm range were 68.9% for OSep and 72.3% for SSep, both considerably higher than that of RawSep (52.5%). These results indicate that the modification treatments primarily optimized the mesoporous structure. Such improvements provide a more favorable spatial framework for asphalt molecule adsorption and the suppression of light component volatilization. However, whether this microstructural optimization can be effectively translated into macroscopic performance enhancement requires further verification through asphalt modification experiments.

### 3.2. Selection of Sepiolite Type

#### 3.2.1. Comparison of Physical Properties

**Figure 3:**
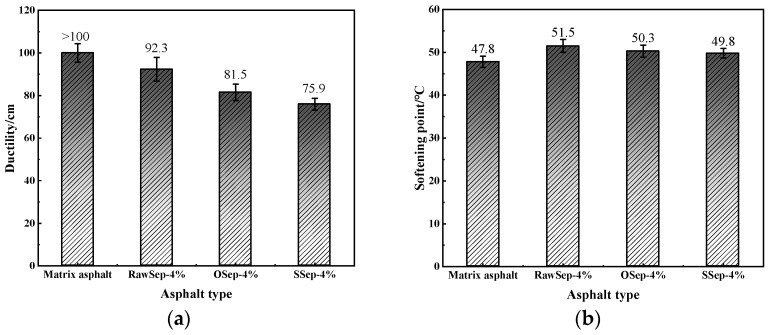
Physical properties of asphalt with different sepiolite types: (**a**) ductility; (**b**) softening point.

Based on previous research experience and preliminary test results, a sepiolite dosage of 4% was selected as the reference level to compare the physical properties of asphalt binders modified with RawSep, OSep, and SSep. The results are illustrated in Figure 3.

As shown in Figure 3, the ductility at low temperature of the RawSep-modified asphalt was 92.3 cm, which, although lower than that of the matrix asphalt, was significantly higher than that of the OSep- and SSep-modified asphalts. This indicates that RawSep exhibited better interfacial compatibility with the asphalt binder, thereby avoiding excessive deterioration of its low-temperature deformation capacity. Regarding the softening point, the RawSep-modified asphalt reached 51.5 °C, an increase of 3.7 °C compared with the matrix asphalt, and higher than the improvements observed for OSep and SSep. This result demonstrates that RawSep provided a more pronounced enhancement of the high-temperature stability of asphalt. Combined with the previous findings, it can be inferred that although OSep and SSep possess larger specific surface areas and pore volumes, the organic functional groups or surfactants introduced during modification may form a coating layer on the sepiolite surface. This layer could hinder the physical adsorption between sepiolite and asphalt molecules and weaken the formation of the fibrous reinforcement network. This phenomenon suggests that the optimization of sepiolite microstructure must be coordinated with the compatibility of the asphalt matrix; merely increasing the specific surface area does not necessarily translate into macroscopic performance improvement.

#### 3.2.2. Comparison of Rheological Properties

**Figure 4:**
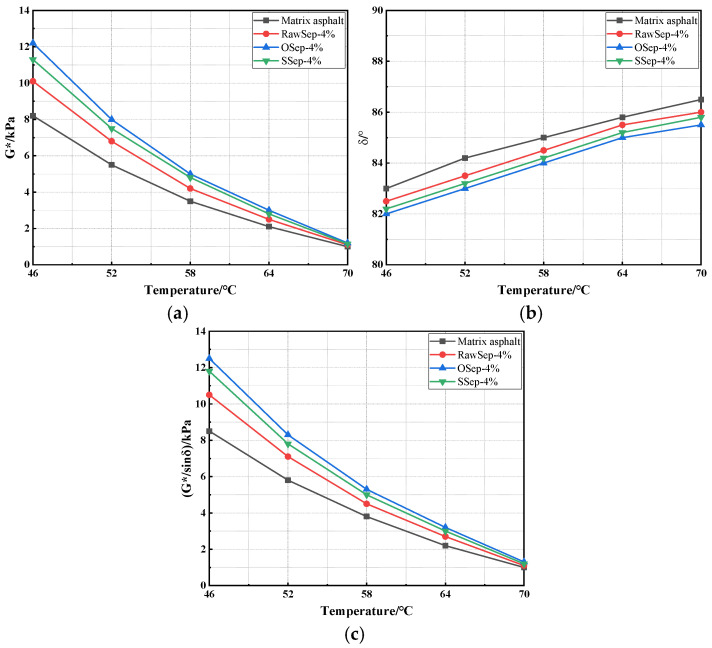
DSR test results of different asphalts: (**a**) G*; (**b**) δ; (**c**) G*/sinδ.

**Figure 5:**
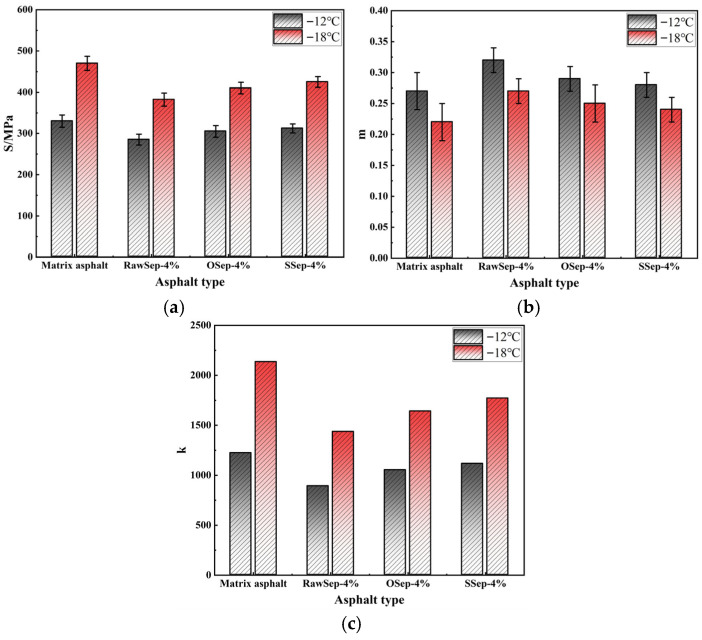
BBR test results of different asphalts: (**a**) S; (**b**) m; (**c**) k.

To comprehensively evaluate the influence of sepiolite type on the high-temperature rheological behavior of asphalt, DSR tests were conducted over the full temperature range of 46 °C to 70 °C [46]. The key rheological parameters—including G*, δ, and G*/sinδ—were extracted for comparison, as shown in Figure 4. In addition, the BBR test results in Figure 5 were analyzed in combination to comprehensively assess the compatibility between sepiolite and asphalt [47].

As shown in Figure 4 and Figure 5, the high-temperature rheological characteristics of asphalt binders exhibit distinct variations depending on the type of sepiolite. At 46 °C, the G* values of the OSep-, SSep-, and RawSep-modified asphalts were 12.2 kPa, 11.3 kPa, and 10.1 kPa, respectively—all markedly higher than the matrix asphalt. As the temperature increased, G* for all samples showed a nonlinear and rapid decline. For OSep-modified asphalt, G* decreased by 33% at 52 °C, 37.5% at 58 °C, and 40% at 64 °C, approaching 1.2 kPa at 70 °C. Throughout the temperature range, the rate of decline in G* for all modified asphalts was slower than that of the matrix asphalt. The trend of G*/sinδ was consistent with that of G*. For instance, the rutting factor of SSep-modified asphalt was 12.5 kPa at 46 °C and remained at 1.3 kPa at 70 °C, consistently higher than that of the matrix asphalt at the same temperatures. In terms of δ, all asphalts exhibited values between 82° and 83° at 46 °C, gradually increasing with temperature. At 70 °C, the phase angle of the matrix asphalt reached 86.5°, while those of the modified asphalts were generally 0.5–1° lower, with the OSep-modified asphalt showing the smallest δ. This indicates a higher proportion of elastic components and superior resistance to viscous deformation at elevated temperatures.

Regarding low-temperature performance, the matrix asphalt exhibited the S of 330 MPa, the m of 0.27, and the k of 1222 at −12 °C; at −18 °C, the corresponding values were S = 470 MPa, m = 0.22, and k = 2136. The OSep- and SSep-modified asphalts showed moderate improvements, with k values of 1052 and 1114 at −12 °C, respectively. The RawSep-modified asphalt, however, demonstrated the most pronounced low-temperature advantages: at −12 °C, S = 285 MPa, m = 0.32, and k = 890; at −18 °C, S = 382 MPa, m = 0.27, and k = 1435. These results indicate superior crack resistance and stress relaxation capacity at low temperatures. Overall, while OSep-modified asphalt exhibited outstanding high-temperature rheological performance, RawSep-modified asphalt achieved a better balance between high-temperature stability and low-temperature cracking resistance. This comprehensive performance alignment makes RawSep more suitable for asphalt pavement applications requiring full-temperature-range adaptability. Consequently, RawSep was selected as the primary modifier for subsequent experiments.

### 3.3. Rheological Properties of Sepiolite-Modified Asphalt

To accurately elucidate the regulatory mechanism of RawSep dosage on the rheological behavior of asphalt, RawSep—previously identified as the optimal modifier—was adopted as the core modification agent. Five dosage gradients (0%, 2%, 4%, 6%, and 8%) were designed. The investigation focused on four key performance dimensions: high-temperature rutting resistance, resistance to permanent deformation, low-temperature cracking resistance, and fatigue damage tolerance. By analyzing parameter variation trends in conjunction with engineering performance requirements, the influencing characteristics of sepiolite dosage on the rheological properties of asphalt were systematically evaluated.

#### 3.3.1. High-Temperature Rutting Resistance

**Figure 6:**
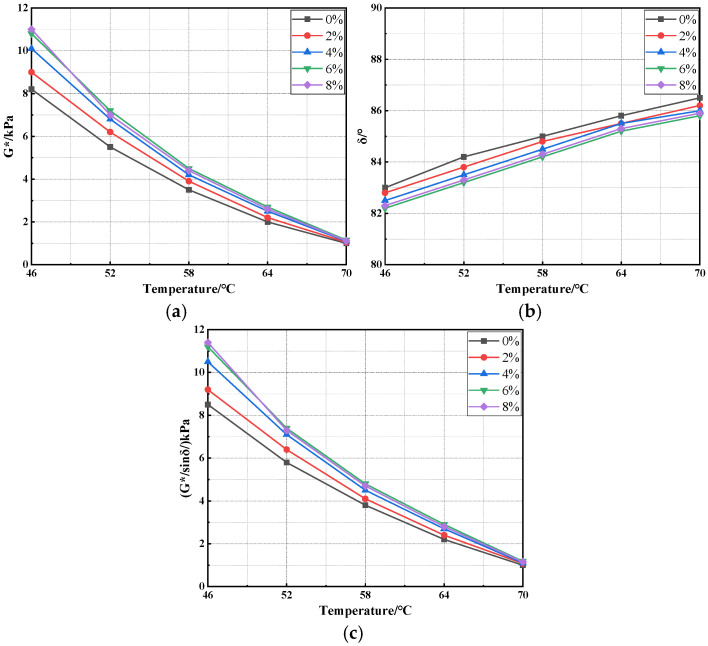
High-temperature performance of RawSep-modified asphalt: (**a**) G*; (**b**) δ; (**c**) G*/sinδ.

To further evaluate the effect of RawSep dosage on the high-temperature rutting resistance of asphalt, DSR tests were conducted on modified asphalt samples with different dosage levels. The results are presented in Figure 6.

As shown in Figure 6, the variation of the G* with RawSep dosage exhibited a “rapid increase followed by stabilization” trend across the entire temperature range. At 46 °C, the G* of the matrix asphalt was 8.2 kPa. When the RawSep dosage increased to 2%, G* rose to 9.0 kPa; at 4%, it reached 10.1 kPa; at 6%, it further increased to 10.8 kPa; and at 8%, it slightly rose to 11.0 kPa, indicating that the growth rate narrowed significantly beyond the 4–6% range. A similar trend was observed at 58 °C, where the matrix asphalt had a G* of 3.5 kPa; at 4% dosage, G* increased to 4.2 kPa, and at 8%, to 4.4 kPa, suggesting that higher dosages led to a plateau in stiffness growth. The δ showed a slight decreasing trend with increasing RawSep content. At 46 °C, δ decreased from 83.0° for the matrix asphalt to 82.5° at 4%, and to 82.3° at 8%, indicating that the addition of RawSep slightly increased the proportion of elastic components, although its influence on δ was weaker than on G*.

The G*/sinδ, a key parameter for evaluating high-temperature rutting resistance, exhibited variation patterns consistent with those of G*. At 46 °C, the rutting factor increased from 8.5 kPa (matrix asphalt) to 9.2 kPa at 2%, 10.5 kPa at 4%, 11.2 kPa at 6%, and 11.4 kPa at 8%. At 70 °C, the matrix asphalt had a rutting factor of 1.0 kPa, which increased to 1.1 kPa at 4% and 1.15 kPa at 8%. These results suggest that at low dosages, RawSep particles are sparsely distributed, failing to form an effective physical skeleton and thus offering limited enhancement of rutting resistance. When the dosage reaches 4–6%, RawSep fibers interweave to form a continuous network, significantly restricting the viscous flow of asphalt molecules and resulting in a marked increase in the rutting factor. At dosages ≥ 8%, RawSep tends to agglomerate, weakening the network structure and leading to internal heterogeneity within the asphalt, which causes performance improvement to plateau. Therefore, the 4–6% range can be identified as the optimal dosage interval for enhancing the high-temperature rutting resistance of RawSep-modified asphalt.

#### 3.3.2. Resistance to Permanent Deformation

**Figure 7:**
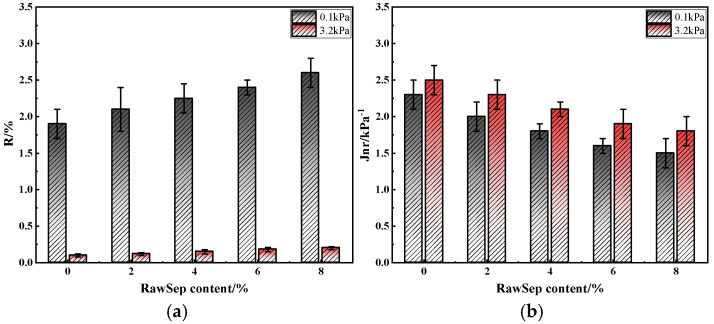
MSCR test results of different asphalts: (**a**) R; (**b**) Jnr.

To further characterize the effect of RawSep dosage on the permanent deformation resistance of asphalt, MSCR tests were conducted on the modified asphalt samples [48]. The R and Jnr were calculated under stress levels of 0.1 kPa and 3.2 kPa, respectively. The results are shown in Figure 7.

As shown in Figure 5, at 0.1 kPa, the R increased steadily with the increase in RawSep dosage, while the Jnr decreased continuously. For the matrix asphalt, R was 1.9% and Jnr was 2.3 kPa^−1^. When the RawSep dosage was 2%, R rose to 2.1%, and Jnr decreased to 2.0 kPa^−1^. At 4%, R reached 2.25% and Jnr dropped to 1.8 kPa^−1^. At 6%, R increased further to 2.4% and Jnr fell to 1.6 kPa^−1^. When the dosage reached 8%, R slightly increased to 2.6%, while Jnr decreased to 1.5 kPa^−1^. Under the 3.2 kPa high-stress condition, the variation trends were consistent with those under low stress, although the absolute values of R and Jnr differed markedly. The matrix asphalt exhibited an R of 0.1% and Jnr of 2.5 kPa^−1^. At 4% RawSep, R increased to 0.15% and Jnr decreased to 2.1 kPa^−1^. At 8%, R reached 0.2% and Jnr declined to 1.8 kPa^−1^.

Notably, the most significant reduction in Jnr occurred at the 4–6% dosage range. Under low stress, Jnr decreased from 1.8 kPa^−1^ to 1.6 kPa^−1^, and under high stress, from 2.1 kPa^−1^ to 1.9 kPa^−1^. When the dosage increased from 6% to 8%, the decrease became less pronounced—Jnr dropped by 6.25% under low stress and 5.26% under high stress. These findings demonstrate that RawSep dosages within the 4–6% range can effectively form a reinforcing skeletal structure that suppresses the permanent deformation of asphalt, while avoiding the agglomeration and diminishing marginal improvement associated with higher dosages. Therefore, within both light- and heavy-load conditions, the 4–6% dosage range provides an optimal balance between permanent deformation resistance and economic feasibility.

#### 3.3.3. Low-Temperature Cracking Resistance

**Figure 8:**
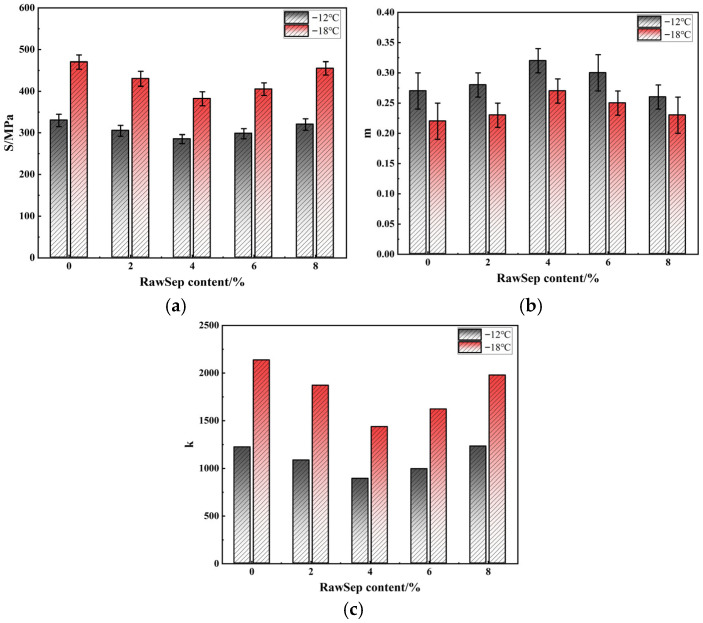
Low-temperature performance of RawSep-modified asphalt: (**a**) S; (**b**) m; (**c**) k.

To further evaluate the influence of RawSep dosage on the low-temperature cracking resistance of asphalt, BBR tests were conducted on the modified asphalt samples. The parameters of S, m, and k were obtained at different test temperatures. The results are presented in Figure 8.

As shown in Figure 8, the influence of RawSep dosage on the low-temperature performance of asphalt followed a “first optimization, then deterioration” trend. At −12 °C, the matrix asphalt exhibited an S value of 330 MPa, a m of 0.27, and a k of 1222. When the RawSep dosage reached 2%, S decreased to 305 MPa, m increased to 0.28, and k declined to 1085, indicating a slight improvement in low-temperature performance. At 4%, S further decreased to 285 MPa, m increased to 0.32, and k dropped to 890, suggesting the best overall cracking resistance. When the dosage increased to 6%, S rose to 298 MPa, m fell to 0.30, and k increased to 993, indicating a partial loss of improvement. At 8%, S increased to 320 MPa, m dropped to 0.26, and k rose to 1231, showing a clear deterioration in low-temperature performance. At −18 °C, similar trends were observed, though parameter fluctuations became more pronounced under extreme cold conditions. The matrix asphalt exhibited S was 470 MPa, m was 0.22, and k was 2136. When the RawSep dosage was 4%, S decreased to 382 MPa, m increased to 0.27, and k decreased to 1435, showing the most significant improvement. However, at 8%, S rose to 455 MPa, m slightly increased to 0.23, and k reached 1978, indicating that the performance advantage almost disappeared. These results suggest that at low dosages, RawSep is insufficiently dispersed and cannot effectively alleviate the brittleness of asphalt at low temperatures. At a 4% dosage, RawSep disperses uniformly within the asphalt matrix, where its physical filling effect reduces brittleness and its fibrous structure enhances stress relaxation capacity. When the dosage reaches 6% or higher, RawSep tends to agglomerate, forming rigid clusters that act as stress concentration sites under low-temperature conditions, thereby worsening brittleness. Consequently, a 4% dosage was identified as the optimum level for achieving the best low-temperature cracking resistance of RawSep-modified asphalt.

#### 3.3.4. Fatigue Damage Resistance

**Figure 9:**
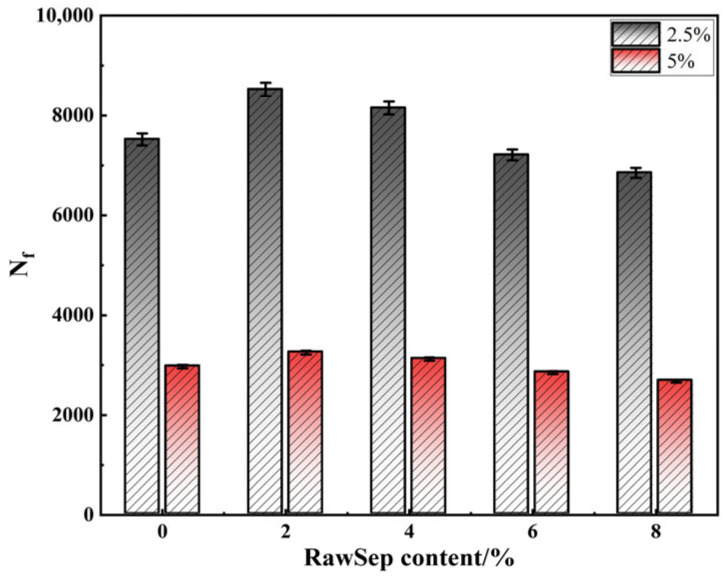
LAS test results of different asphalts.

To further evaluate the influence of RawSep dosage on the fatigue damage resistance of asphalt, LAS tests were performed on the modified asphalt samples [49]. The corresponding Nf values were obtained under different strain levels. The results are presented in Figure 9.

As shown in Figure 9, under the 2.5% strain condition, the Nf of the asphalt samples exhibited a “first increase, then decrease” trend with increasing RawSep dosage. The matrix asphalt had an Nf of 7520 cycles. When the dosage was 2%, Nf increased to 8520 cycles, indicating the best fatigue performance. At 4%, Nf decreased slightly to 8150 cycles; at 6%, it dropped to 7210 cycles, and at 8%, it further declined to 6850 cycles. A similar pattern was observed under the 5.0% strain condition, though the absolute Nf values were lower and the reduction trend more pronounced. The matrix asphalt showed an Nf of 2970 cycles; when 2% RawSep was added, Nf increased to 3250 cycles, then decreased to 3120 cycles at 4%, and further dropped to 2680 cycles at 8%. The underlying mechanism can be explained as follows: at low dosages, RawSep acts as a stress-dissipation medium, delaying the accumulation of viscoelastic damage in the asphalt network. During cyclic loading, RawSep fibers bear part of the applied stress, reducing local stress concentrations within the asphalt matrix and thus extending the Nf. However, at higher dosages, RawSep particles tend to agglomerate, forming heterogeneous rigid clusters that serve as initiation sites for damage. Under repeated loading, microcracks easily develop at the interface between the agglomerates and the asphalt matrix, which then propagate rapidly along the clusters, accelerating fatigue failure. Therefore, the 2–4% dosage range represents the optimal interval for improving the fatigue damage resistance of RawSep-modified asphalt. Among these, the 2% dosage yields the best fatigue performance, while 4% maintains superior fatigue life compared with the matrix asphalt and simultaneously provides balanced high- and low-temperature performance.

### 3.4. Analysis of Fume Components in RawSep-Modified Asphalt

Based on the previously identified optimal RawSep dosage, the GC–MS technique was employed to quantitatively evaluate the smoke-suppression effect of RawSep-modified asphalt. The main fume components were quantified using the peak area normalization method, and the results are summarized in Table 4.

As shown in Table 4, the fume from the matrix asphalt primarily consisted of aliphatic hydrocarbons—mainly C8–C16 alkanes—and aromatic hydrocarbons, including mono- and bicyclic compounds such as naphthalene and phenanthrene. Together, these two groups accounted for 80.7% of the total organic pollutants. When 2% RawSep was added, the aliphatic fraction decreased to 37.8%, and the aromatic fraction dropped to 33.6%, indicating the initial manifestation of smoke-suppression effects. At the 4% dosage, the aliphatic component further decreased to 31.2% and the aromatic component to 25.8%, while no polycyclic aromatic hydrocarbons (PAHs) such as benzo[a]pyrene were detected. This demonstrates that the physical adsorption and chemical inhibition effects of RawSep fibers were both significant. However, when the dosage increased to 6–8%, the relative contents of both groups slightly rebounded—at 6%, the aliphatic and aromatic fractions were 33.5% and 28.1%, respectively, and at 8%, 35.7% and 30.3%. This suggests that excessive RawSep leads to particle agglomeration, reducing available adsorption sites and thereby lowering fume-suppression efficiency. In summary, a 4% RawSep dosage provided the optimal suppression effect on characteristic pollutants in asphalt fumes, effectively reducing the release of aliphatic and aromatic hydrocarbons. This demonstrates the potential of RawSep modification as a viable approach for fume control during asphalt production and construction.

### 3.5. Microscopic Interaction Mechanism Between RawSep and Asphalt

#### 3.5.1. FTIR Analysis

**Figure 10:**
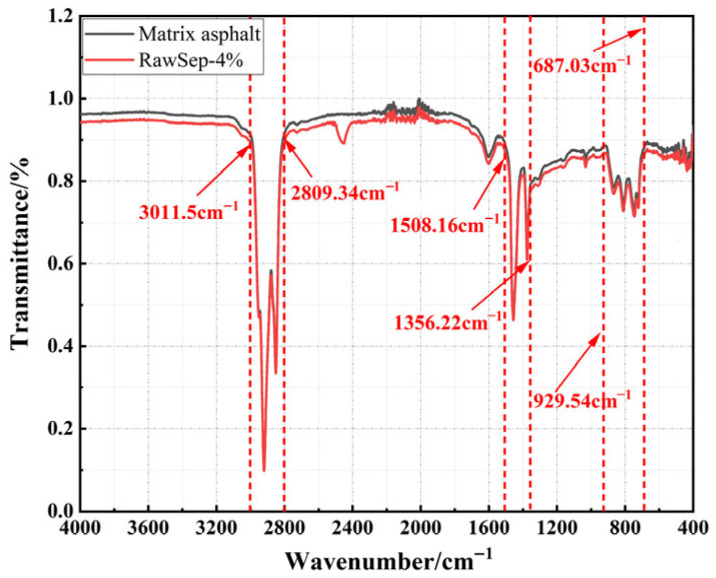
FTIR test results of RawSep-modified asphalt.

To elucidate the interaction mechanism between asphalt and RawSep within the modified binder, an FTIR test was conducted to characterize the asphalt before and after modification [50]. The results are presented in Figure 10.

As shown in Figure 10, noticeable differences are observed between the FTIR spectra of the matrix asphalt and the RawSep-modified asphalt in the range of 3011.5–2809.34 cm^−1^. The characteristic peaks of the modified asphalt in this region show distinct variations, indicating that RawSep interacts with the hydrogen-containing functional groups of aromatic and aliphatic hydrocarbons in asphalt. This interaction may occur through physical adsorption or the influence of surface functional groups on these molecular structures, leading to changes in vibrational absorption behavior. As a result, the state of aromatic and aliphatic components in the asphalt is altered, which helps reduce the volatilization of small molecular species. In the range of 1508.16–1356.22 cm^−1^, the characteristic peaks of RawSep-modified asphalt also differ from those of the matrix asphalt, suggesting that the addition of RawSep alters the vibrational environment of aromatic ring structures and aliphatic side chains. This may be attributed to the interaction between RawSep surface groups and asphalt molecules, which stabilizes the aromatic ring structure while affecting the aliphatic side chains, thereby enhancing the structural stability of asphalt molecules. These findings are consistent with earlier conclusions regarding the reduction in pyrolysis products and fume emissions in modified asphalt. In the region of 687.03–929.54 cm^−1^, new characteristic peaks appear in the RawSep-modified asphalt that are absent in the matrix asphalt, indicating that the intrinsic functional groups of RawSep remain preserved after dispersion within the asphalt matrix. These groups play dual roles: on the one hand, they provide physical reinforcement within the asphalt system; on the other, they engage in interfacial interactions with asphalt molecules, thereby improving interfacial bonding and contributing to enhanced macroscopic performance. The absence of new oxidation or esterification peaks further confirms that the interaction between RawSep and asphalt is predominantly physical rather than chemical in nature.

#### 3.5.2. GPC Analysis

To further verify the interaction between RawSep and the asphalt matrix, GPC tests were conducted on the asphalt samples before and after modification [51]. The results are presented in Table 5.

As shown in Table 5, the matrix asphalt contained 38.5% small-molecule components, 42.3% medium-molecule components, and 19.2% large-molecule components, with a polydispersity index (PDI) of 3.25, indicating a relatively broad molecular weight distribution and moderate colloidal stability. In contrast, the RawSep-modified asphalt exhibited a decrease in small-molecule content to 31.8%, an increase in medium-molecule content to 46.5%, and an increase in large-molecule content to 21.7%, while the PDI decreased to 2.82, reflecting a narrower molecular weight distribution and markedly improved colloidal stability. This transformation suggests that the incorporation of RawSep reduces the proportion of small molecules through physical adsorption, while simultaneously promoting the conversion of some small molecules into medium- and large-molecular-weight fractions. This effect may be associated with hydrogen bonding-induced molecular aggregation, which leads to a more compact colloidal structure within the asphalt matrix. From a microscopic perspective, this structural densification supports the improvements in high-temperature deformation resistance and low-temperature cracking resistance, consistent with the rheological test results presented in Section 3.3.

## 4. Conclusions

This study aimed to expand the application of sepiolite in asphalt modification for road engineering by addressing the performance limitations and environmental challenges of conventional asphalt. Through organic and surface modification, the microstructure of sepiolite was optimized, and the most compatible type was selected. Different dosage gradients were designed, and a series of multiscale experiments were conducted to systematically investigate the regulatory effects of sepiolite on asphalt properties and to reveal the underlying microscopic interaction mechanisms. Based on the experimental results, the following conclusions can be drawn:

(1) RawSep exhibited the best compatibility with asphalt. Although the specific surface areas of OSep and SSep increased by 43–45% after modification, the high–low temperature coordination of the modified asphalt decreased by 10–15%, indicating that the optimization of sepiolite microstructure must be coupled with the compatibility of the asphalt matrix.
(2) Sepiolite dosage had a significant impact on the high-temperature rutting and permanent deformation resistance of asphalt. At dosages of 4–6%, the rutting factor increased by 25–30%, resulting in the best high-temperature deformation resistance. Insufficient dispersion at low dosages and particle agglomeration at high dosages both hinder performance improvement.
(3) The 4% RawSep-modified asphalt exhibited the best low-temperature cracking resistance. At this dosage, the creep stiffness decreased by more than 15%. Poor dispersion at low dosages failed to mitigate asphalt brittleness, whereas high dosages caused fiber agglomeration and the formation of rigid particles, which aggravated low-temperature cracking.
(4) Sepiolite at dosages of 2–4% effectively improved the fatigue life of asphalt by 9–13%. The most significant enhancement occurred at 2%, where the stress-dissipation effect of sepiolite delayed damage accumulation. At higher dosages, agglomerated particles acted as stress concentrators, accelerating fatigue failure.
(5) The 4% RawSep-modified asphalt demonstrated the most effective suppression of characteristic pollutants in asphalt fumes. The emissions of aliphatic and aromatic hydrocarbons were significantly reduced, leading to a 12–18% decrease in toxic fume emissions. The porous structure of sepiolite adsorbed 3–5% of light volatile compounds, while its metal oxides inhibited the release of aliphatic and aromatic hydrocarbons.
(6) FTIR and GPC analyses revealed that the interaction between sepiolite and asphalt was dominated by physical adsorption, without chemical functional group recombination. The fibrous network of sepiolite enhanced the structural stability of asphalt, while the adsorption of small- and medium-molecular components optimized the molecular weight distribution. These microstructural effects supported the macroscopic improvements in performance and fume suppression.

Although this study provides basic data support for the development and application of sepiolite-modified asphalt, it still has certain risks and limitations: there are differences between the indoor standard test environment and the complex service conditions in actual engineering, such as extreme temperatures, ultraviolet radiation, and rainwater erosion; the parameters for large-scale sample preparation in the laboratory are difficult to fully replicate in industrial production scenarios; moreover, the research focuses on the performance evaluation of the asphalt matrix, lacking data on the road performance of mixtures, verification of long-term service performance, and analysis of the whole-life cycle economic costs, which may affect the engineering adaptability and promotion feasibility of the results. In the future, in the scientific field, the interface interaction between sepiolite and asphalt molecules, element distribution, and chemical kinetic mechanisms can be further studied through combined AFM and SEM-EDS technologies; the composite synergistic effects of sepiolite with other modifiers can be explored to improve the correlation theory of “microstructure-macroscopic performance”. In the application field, the dosage formula can be optimized for different climate zones to develop special modified asphalt; an environmental protection system of “source inhibition-end purification” can be constructed to promote its pilot application and standardized construction in various road engineering scenarios; meanwhile, its application can be extended to the field of recycled waste asphalt, contributing to the green, low-carbon and circular development of road engineering.

## Figures and Tables

**Table 1:** Technical properties of matrix asphalt.

Index	Unit	Test Value	Requirement
Penetration	0.1 mm	64.2	60.0~80.0
Ductility	cm/15 °C	>100	≥100
Softening point	°C	47.8	≥46.0
Saturates content	%	12.5	
Aromatics content	%	30.2	
Resins content	%	38.8	
Asphaltenes content	%	18.5	

**Table 2:** Chemical composition of raw sepiolite.

Component	SiO_2_	MgO	Al_2_O_3_	Fe_2_O_3_	CaO	K_2_O	Na_2_O	TiO_2_	Others
Content/%	58.01	24.15	1.74	0.56	0.42	0.38	0.24	0.08	14.32

**Table 3:** Microstructural parameters of sepiolite with different modification methods.

Sepiolite Type	BET Specific Surface Area/(m^2^·g^−1^)	Total Pore Volume/(cm^3^·g^−1^)	Average Pore Diameter/nm	Mesopore (2~50 nm) Volume Ratio/%	2~20 nm Pore Volume Ratio/%
RawSep	107.95	0.32	11.8	91.2	52.5
OSep	154.83	0.45	9.2	94.5	68.9
SSep	156.53	0.48	9.5	93.8	72.3

**Table 4:** Effect of RawSep content on relative content of main components in asphalt fume.

RawSep Content	Aliphatic Hydrocarbons/%	Aromatic Hydrocarbons/%	Other Components/%	Polycyclic Aromatic Hydrocarbons Detection
0%	42.5	38.2	19.3	Naphthalene, Phenanthrene, Fluoranthene
2%	37.8	33.6	28.6	Naphthalene, Phenanthrene
4%	31.2	25.8	43.0	-
6%	33.5	28.1	38.4	Naphthalene
8%	35.7	30.3	34.0	Naphthalene, Phenanthrene

**Table 5:** GPC test results.

Sample Type	Low-Molecular-Weight Fraction/%	Medium-Molecular-Weight Fraction/%	High-Molecular-Weight Fraction/%	Number-Average Molecular Weight (Mn)	Weight-Average Molecular Weight (Mw)	Polydispersity Index (PDI)
Matrix asphalt	38.5	42.3	19.2	850	2763	3.25
RawSep-4%	31.8	46.5	21.7	920	2594	2.82

## Data Availability

The original contributions presented in this study are included in the article. Further inquiries can be directed to the corresponding author.
